# The influence of a consumer-wearable activity tracker on sedentary time and prolonged sedentary bouts: secondary analysis of a randomized controlled trial

**DOI:** 10.1186/s13104-018-3306-9

**Published:** 2018-03-22

**Authors:** Robert A. Sloan, Youngdeok Kim, Aarti Sahasranaman, Falk Müller-Riemenschneider, Stuart J. H. Biddle, Eric A. Finkelstein

**Affiliations:** 1 Department of Psychosomatic Internal Medicine, Graduate School of Medical and Dental Sciences, Kagoshima University, Kagoshima, Japan; 2 Texas Tech University, Lubbock, USA; 3 Duke-NUS Medical School, Singapore, Singapore; 4 National University of Singapore, Singapore, Singapore; 5 University of Southern Queensland, Springfield Central, Australia

**Keywords:** Sedentary behavior, Sedentary time, Prolonged sedentary bouts, Consumer-wearable activity tracker, Fitbit, Secondary analysis of an RCT

## Abstract

**Objective:**

A recent meta-analysis surmised pedometers were a useful panacea to independently reduce sedentary time (ST). To further test and expand on this deduction, we analyzed the ability of a consumer-wearable activity tracker to reduce ST and prolonged sedentary bouts (PSB). We originally conducted a 12-month randomized control trial where 800 employees from 13 organizations were assigned to control, activity tracker, or one of two activity tracker plus incentive groups designed to increase step count. The primary outcome was accelerometer measured moderate-to-vigorous physical activity.

**Results:**

We conducted a secondary analysis on accelerometer measured daily ST and PSB bouts. A general linear mixed model was used to examine changes in ST and prolonged sedentary bouts, followed by between-group pairwise comparisons. Regression analyses were conducted to examine the association of changes in step counts with ST and PSB. The changes in ST and PSB were not statistically significant and not different between the groups (*P* < 0.05). Increases in step counts were concomitantly associated with decreases in ST and PSB, regardless of intervention (*P* < 0.05). Caution should be taken when considering consumer-wearable activity trackers as a means to reduce sedentary behavior.

*Trial registration* NCT01855776 Registered: August 8, 2012

**Electronic supplementary material:**

The online version of this article (10.1186/s13104-018-3306-9) contains supplementary material, which is available to authorized users.

## Introduction

Daily life is becoming increasingly sedentary, and working adults are spending more time sitting [1]. Evidence shows that too much sedentary time (ST), and prolonged sedentary bouts (PSB), increases morbidity and premature mortality independent of moderate-to-vigorous physical activity (MVPA) [2, 3]. The Sedentary Behavior Research Network recently defined a bout of ST as a period of uninterrupted ST (≤ 1.5 METs) and a prolonger as someone who accumulates ST in extended continuous bouts [4]. Specifically, the American Diabetes Association recommends individuals break up ST every 30 min with light-intensity activity [5]. Because of the ubiquitous nature and deleterious effects of sedentary lifestyles, public health professionals are seeking practical approaches to decrease ST and PSB [2, 3].

Although MVPA interventions are not generally considered beneficial for reducing ST, a recent meta-analysis reported a moderate effect size for pedometer based MVPA interventions to inadvertently reduce objectively measured daily ST independent of changes in MVPA and steps [6]. Primary limitations of the meta-analysis included no assessment of PSB and short investigation periods. Cochrane reviews have indicated insufficient evidence for interventions regarding sedentary behavior with a critical need for future studies to assess the effectiveness of interventions over the long term using objective measurement of ST and PSB [7, 8].

There is a dearth of evidence regarding the impact of *consumer*-*wearable activity trackers* on ST and PSB [9]. Compared to pedometers, activity trackers have the ability to integrate with information communication technology platforms and provide feedback. We recently conducted a 6-month intervention with a 6-month post intervention follow-up workplace-based RCT (TRIPPA) to investigate the effectiveness of the web-supported Fitbit Zip™ with and without incentive-based step goals to improve MVPA [10]. The intervention groups had more objectively measured MVPA at month 12. We also found self-reported sitting time decreased. However, we did not investigate *objectively* measured ST or PSB. While there is evidence pedometers decrease ST, a practical question emerges from the literature; can use of an activity tracker inadvertently reduce ST and PSB? As an extension to our original investigation, this secondary data analysis examined the effectiveness of the original interventions to reduce ST and PSB.

## Main text

### Methods

TRIPPA was a 4-arm, 6-month randomized controlled trial with a 6-month post-intervention follow-up period, conducted in 13 private and government organisations in Singapore. The majority of job types were sedentary in nature. We randomly assigned participants (n = 800) to one of four study groups: No Fitbit Zip™ or incentive (Control, n = 201), Fitbit Zip™ + no incentive-based step goal (Fitbit, n = 203), Fitbit Zip™ + charity incentive-based step goal (Charity, n = 199), and Fitbit Zip™ + cash incentive-based step goal (Cash, n = 197) (see Additional file 1). A detailed description of the methods and protocols used in this investigation was already published, new additions or variations have been stated [10, 11]. The institutional review board at the National University of Singapore approved the study protocol, and the study is registered at ClinicalTrials.gov, Number NCT01855776.

All outcomes were measured for participants in all groups via sealed accelerometers [ActiGraph triaxial GT-3x + accelerometer (ActiGraph, Pensacola, FL, USA)] at baseline, 6, and 12 months. We instructed participants to wear the accelerometer for at least 10 waking hours on each day for 7 consecutive days during each assessment interval. Data were downloaded in 60-s epochs, and wear time checks were performed using the ActiLife software (ActiGraph, Pensacola, FL, USA). Accelerometer non-wear time was defined using the Choi et al. [12] algorithm as consecutive zero vector magnitude (VM) counts for at least 90 min (window 1), allowing for short time intervals with non-zero counts lasting up to 2 min if no counts were detected during both the 30 min (window 2) upstream and downstream from that interval. Any non-zero counts except the allowed short intervals were considered wear time [12, 13]. We processed accelerometer data in R (version 3.1.2; R Core Team, Vienna, Austria) using the accelerometery package for triaxial accelerometers to obtain standard variables based on vector magnitude counts [14]. We defined ST using the Matthews’ cut-point of < 100 counts per min [15]. Based on the American Diabetic Association Guideline [5], we defined PSB as a total of 30 min or more at the Matthews’ cut-point [16].

We performed secondary data analyses [17] to assess the impact of activity tracker use on ST and 30-min PSB via accelerometery at 6, and 12 months. The percentages (%) of ST and PSB were calculated over total wear time for each monitoring day and averaged across valid days for each measurement point. Daily steps were averaged across valid days and were least square adjusted to the average wear time.

The sample size was previously determined to achieve a statistical power of .80 to detect 0.35 *SD* difference in mean moderate-to-vigorous MVPA bout min per week (≈ 30 MVPA bout min per week) between groups, assuming an intraclass correlation coefficient of 0.075 [10]. For this study, the participants who provided valid accelerometer data for at least 4 days, including 1 weekend day, were considered valid at each measurement point, and all analyses were based on an intent-to-treat principle. A general linear mixed model was used to examine the differences in changes in ST and PSB between groups after controlling for the covariates of age, gender, and ethnicity. The model was best fitted with an unstructured variance–covariance organisation for the repeated measures based on Akaike and Bayesian information criteria. The restricted maximum likelihood estimation method was used to account for missing data under the assumption of missing at random. Also, a series of multiple regression analyses were conducted to examine the parallel associations between step counts with ST and PSB across three measurement points after controlling for study covariates. Preliminary analyses showed that there were no significant moderating effects on the relationship between step counts and ST or PSB. The analyses were based on the pooled sample across groups. The parallel associations were examined by regressing changes in step counts against changes in ST and PSB between each pair of measurement points. For ease of interpretation, the unit of step counts was converted to 100 steps/day. The missing data were handled using the Markov chain Monte Carlo multiple imputation method, and the pooled estimates were obtained from 20 imputed datasets for each regression analysis using Rubin’s rule [18]. SAS v9.4 (SAS Institute, Cary, NC) was used for all analyses and a family-wise alpha level of .05 was used for statistical significance.

### Results

Additional file 1 presents the number of valid participants over times and baseline measures of outcome variables by groups. Of the initial sample of 800, more than 90% of study participants provided valid accelerometer data for at least 4 days, including 1 weekend day at baseline, and the retention rates were in the range of 86.2–93.4% and 73.9–81.1% at 6 and 12 months, respectively, across groups. The participants spent more than 70% of their total waking hours in ST, and ~ 25% of waking hours were spent in PSB.

The results of general linear mixed analyses examining the changes in ST and PSB over time by groups are presented in Table 1. Overall, the control group showed an increase trend in total ST (P < .05) in which ST at M6 (*M* = 72.05%; *SE* = 0.51) and M12 (*M* = 71.99; *SE* = 0.53) were significantly higher than the baseline (*M* = 70.96%; *SE* = 0.50). Similarly, there were increased trends in PSB across groups; however, these trends were not statistically significant over time. Further analyses of between-group pairwise comparisons also revealed that changes in ST and PSB between each time point were not significantly different between the groups (Table 2). Table 3 presents the associations of step counts with ST and PSB. We found significant and negative parallel associations in which an average increase of 100 steps per day between each time point was associated with decreased change in an average of 15–16% (95% CI ranges 14–17%) for ST and 12% (95% CI ranges 8–15%) for PSB, respectively.

**Table 1:** Changes in objectively measured sedentary behavior across three measurement points

	Baseline	M6	M12	P for linear-trends
Total ST (%/day)				
Control	70.96 (0.50)^a,b^	72.05 (0.51)	71.99 (0.53)	.021
Fitbit	70.88 (0.49)	71.38 (0.51)	70.91 (0.54)	.940
Charity	70.42 (0.50)	71.78 (0.53)	71.51 (0.56)	.839
Cash	70.75 (0.49)	70.74 (0.50)	70.64 (0.54)	.798
30-min PSB (%/day)				
Control	26.53 (0.79)	28.01 (0.83)	27.74 (0.83)	.108
Fitbit	25.98 (0.77)	27.24 (0.82)	26.16 (0.84)	.812
Charity	25.88 (0.80)	26.51 (0.85)	26.60 (0.87)	.357
Cash	24.81 (0.77)	26.39 (0.81)	25.49 (0.84)	.374

*ST* sedentary time, *PSB* prolonged sedentary bouts. Values are least-square mean (SE) estimated from the linear mixed models adjusting for study covariates (age, gender, and ethnicity)

^a^Significantly different from M6 at Bonferroni adjusted *α* level of .0125. ^b^ Significantly different from M12 at Bonferroni adjusted *α* level of .0125

**Table 2:** Between-group comparisons changes in the percentage of sedentary behavior

	Control–Fitbit	Control–cash	Control–charity	Fitbit–cash	Fitbit–charity	Cash–charity
Δ (month 6 − baseline)						
Total ST (%/day)	0.74 (− 0.62, 2.09)	1.25 (− 0.08, 2.58)	0.68 (− 0.69, 2.05)	0.52 (− 0.81, 1.84)	− 0.06 (− 1.42, 1.31)	0.57 (− 1.92, 0.77)
30-min PSB (%/day)	0.58 (− 1.74, 2.90)	0.66 (− 1.62, 2.95)	1.19 (− 1.15, 3.54)	0.09 (− 2.19, 2.36)	0.62 (− 1.71, 2.95)	0.53 (− 1.77, 2.83)
Δ (month 12 − baseline)						
Total ST (%/day)	0.91 (− 0.65, 2.46)	1.19 (− 0.37, 2.75)	0.54 (− 1.04, 2.12)	0.28 (− 1.29, 1.85)	− 0.37 (− 1.96, 1.23)	− 0.65 (− 2.25, 0.95)
30-min PSB (%/day)	1.21 (− 1.35, 3.78)	1.62 (− 0.95, 4.19)	0.85 (− 1.75, 3.45)	0.40 (− 2.19, 2.99)	− 0.36 (− 2.99, 2.26)	− 0.77 (− 3.40, 1.87)
Δ (month 12 − month 6)						
Total ST (%/day)	0.68 (− 0.83, 2.18)	0.47 (− 1.04, 1.97)	0.36 (− 1.17, 1.89)	− 0.21 (− 1.74, 1.32)	− 0.32 (− 1.87, 1.24)	− 0.11 (− 1.67, 1.45)
30-min PSB (%/day)	1.04 (− 1.43, 3.50)	1.20 (− 1.26, 3.67)	0.44 (− 2.07, 2.95)	0.17 (− 2.33, 2.67)	− 0.60 (− 3.14, 1.95)	− 0.76 (− 3.31, 1.78)

*ST* sedentary time, *PSB* prolonged sedentary bouts. The values are presented as mean and 95% confidence intervals adjusted for multiple pairwise comparisons using the Tukey’s method. The linear mixed model was used for analyses after adjusting for study covariates (age, gender, and ethnicity)

**Table 3:** Associations of step counts with sedentary behavior over three measurement points

	Total ST (%)	30-min PSB (%)
Δ (M6 − baseline)^a^	− 0.15** (− 0.17, − 0.14)	− 0.12** (− 0.15, − 0.09)
Δ (M12 − baseline)^a^	− 0.16** (− 0.17, − 0.14)	− 0.12** (− 0.15, − 0.08)
Δ (M12 − M6)^a^	− 0.15** (− 0.17, − 0.14)	− 0.12** (− 0.15, − 0.08)

*ST* sedentary time, *PSB* prolonged sedentary bouts. Values are unstandardized regression coefficient and 95% confidence intervals estimated from the pooled analyses of 20 imputed datasets

^a^The parallel associations of changes in step counts (unit: Δ100 steps/day) with changes in sedentary times (Δ%/day) between two measurement points. The estimates in this row are interpreted as the predicted changes in sedentary times (%/day) between two measurement points for 100 incremental changes in steps/day between two measurement points

* *P *< .001, ** *P *< .01

### Discussion

Consumer-wearable activity trackers are reportedly the number-one worldwide fitness trend [19]. In our investigation, the use of an activity tracker did not reduce objectively measured ST or PSB among healthy working adults. Meanwhile, we found that an activity tracker may prevent an increase in sedentary behavior and increasing steps independently associates with potential decreases in ST and PSB. To our knowledge, this is the first evaluation of a large scale RCT to objectively assess the practical effectiveness of an activity tracker on ST and PSB over an extended period.

A recent meta-analysis investigated pedometer use and objectively measured ST in unhealthy older adult populations [6]. Similar to our current investigation, each of the RCTs in the meta-analysis originally focused on increasing MVPA. The overall findings indicated the mere use of a pedometer could generate significant reductions in objectively measured ST. The authors concluded the findings provided a “widespread recommendation and adoption of step counter use in health promotion programs.” We showed that an activity tracker was ineffective for reducing objectively measured ST and PSB, regardless of the intervention type. The meta-analysis also showed reductions in ST were independent of steps, yet we found an independent inverse association between step count with ST and PSB.

It may be that other salient features need to be added to activity trackers, such as acute reminders to reduce ST and PSB. Biddle et al. conducted a robust 12-month RCT and found reminder vibrations to be ineffective for reducing objectively measured ST [20]. A recent pilot study in a small group of college students conducted over several weeks suggested that a research grade activity tracker providing only vibration feedback could effectively reduce PSB by ~ 20%. The control group with no vibration reminder had ~ 10% reduction in PSB with no significant effect. Neither group had changes in MVPA or ST. The advantage of the study compared to our study was the research grade wearable could detect all forms of light activity, including standing time.

Lastly, while we did not focus on the role of financial incentives in our current evaluation, it is important to discuss. Ball et al. conducted a 4-month pilot study investigating the effects of incentives (clothing, recipe books, and store gift vouchers) for increasing self-reported MVPA and reducing ST among inactive adults 40–65 years. Similar to our study, a Fitbit One™ wearable activity tracker was used by participants to sync to a website and provide awards [21]. Motivational interviewing and text messaging were also used to promote behavior change. At the end of the intervention, self-reported ST decreased by ~ 3 h/day. A decrease in self-reported sitting time with cash incentives was found in our original study but not in objectively measured ST in this current investigation [10]. The difference may be accounted for by self-report bias compared to objectively measured ST [22]. Our findings call into question the use of activity trackers as a panacea for reducing ST as suggested by Qiu and colleagues’ meta-analysis [6]. Nonetheless, our findings along with the limited literature generated potential new areas of exploration. Particularly, more development and investigation with use of consumer-wearable activity trackers that provide salient feedback and nuanced approaches to reduce ST and PSB is warranted [23, 24].

### Conclusion

Since sedentary behavior permeates daily life, unique and creative intervention approaches should be examined. More emphasis on developing and evaluating the effectiveness of *sedentary trackers* rather than activity trackers is called for. Future long-term studies should investigate combinations of workplace strategies, emerging technologies, behavioral techniques, and the role of incentives to reduce sedentary behavior.

### Limitations

- Secondary analysis of data from a RCT originally designed to use incentive-based step goals to increase MVPA.
- There may be unclear bias due to the lack of compliance for wearing the Fitbit Zip™ among experimental groups.
- The consumer-wearable activity tracker used in this study was waist worn which may limit the generalization of the results compared to other types of trackers, i.e. wrist worn.
- This study population may limit the generalization of results.
- Missing data addressed in this study did not include a degree to which the participants were compliant with study protocols for wearing the Fitbit Zip™.
- Assessing ST with wearable technology does not allow for the assessment of time in specific domains/behaviors, such as TV viewing and computer use. This may be important, as some sedentary behaviors have been shown to have stronger links to adverse health outcomes than others [25].

## Additional file

**Additional file 1.** Demographics. Demographic characteristics of study participants by intervention groups. Accelerometer profiles and baseline measures of outcome variables by intervention groups.

## Abbreviations

ST: sedentary time
PSB: prolonged sedentary bouts
MVPA: moderate-to-vigorous physical activity
RCT: randomized control trial
TRIPPA: TRial of Economic Incentives to Promote Physical Activity
