# Supplementary material for: The influence of a consumer-wearable activity tracker on sedentary time and prolonged sedentary bouts: secondary analysis of a randomized controlled trial
Source: BMC Res Notes. 2018 Mar 22;11:189. doi: 10.1186/s13104-018-3306-9 (PMC5863802; doi:10.1186/s13104-018-3306-9)
Supplement: Supplementary file 1 — Additional file 1. Demographics. Demographic characteristics of study participants by intervention groups. Accelerometer profiles and baseline measures of outcome variables by intervention groups. [file 13104_2018_3306_MOESM1_ESM.docx]

**Additional File.1**

**Demographic Characteristics of Study Participants by Intervention Groups**

|  | Control  (*n*=201) | Fitbit Only  (*n*=203) | Charity  (*n*=199) | Cash  (*n*=197) |
| --- | --- | --- | --- | --- |
| Age (years), Mean (*SD*) | 35.6 (8.6) | 35.4 (8.3) | 35.5 (8.6) | 35.5 (8.4) |
| Gender, *n* (%) |  |  |  |  |
| Male | 89 (44.3) | 103 (50.7) | 94 (47.2) | 84 (42.6) |
| Female | 112 (55.7) | 100 (49.3) | 105 (52.8) | 113 (57.4) |
| Ethnicity, *n* (%) |  |  |  |  |
| Chinese | 138 (68.7) | 133 (65.5) | 145 (72.9) | 132 (67.0) |
| Malay | 10 (4.9) | 13 (6.4) | 5 (2.5) | 8 (4.1) |
| Indian | 30 (14.9) | 26 (12.8) | 17 (8.5) | 32 (16.2) |
| Other | 19 (9.5) | 26 (12.8) | 27 (13.6) | 24 (12.2) |
| Not declared | 4 (2.0) | 5 (2.5) | 5 (2.5) | 1 (0.5) |
| Education, *n* (%) |  |  |  |  |
| High school or lower | 12 (5.9) | 9 (4.4) | 7 (3.5) | 11 (5.6) |
| Some college | 31 (15.4) | 27 (13.3) | 21 (10.6) | 28 (14.2) |
| College graduate | 98 (48.8) | 100 (49.3) | 100 (50.3) | 80 (40.6) |
| Postgraduate | 56 (27.9) | 58 (28.6) | 60 (30.1) | 73 (37.1) |
| Other | 0 (0.0) | 3 (1.5) | 6 (3.0) | 4 (2.0) |
| Not declared | 4 (2.0) | 6 (2.9) | 5 (2.5) | 1 (0.5) |
| Monthly personal income, *n* (%) |  |  |  |  |
| <$5,000 | 103 (51.2) | 93 (45.8) | 92 (46.3) | 92 (46.7) |
| $5,000 - $9,999 | 41 (20.4) | 46 (22.7) | 43 (21.6) | 37 (18.8) |
| ≥$10,000 | 12 (6.0) | 15 (7.4) | 8 (4.0) | 15 (7.6) |
| Prefer not to say | 40 (19.9) | 43 (21.3) | 50 (25.1) | 52 (26.4) |
| Don’t know | 1 (0.5) | 1 (0.5) | 1 (0.5) | 0 (0.0) |
| Not declared | 4 (2.0) | 5 (2.5) | 5 (2.5) | 1 (0.5) |
| Monthly household income, *n* (%) |  |  |  |  |
| <$5,000 | 52 (25.9) | 49 (24.1) | 49 (24.6) | 53 (26.9) |
| $5,000 - $9,999 | 56 (27.9) | 57 (28.1) | 49 (24.6) | 41 (20.8) |
| ≥$10,000 | 26 (12.9) | 29 (14.3) | 30 (15.6) | 30 (15.2) |
| Prefer not to say | 46 (22.9) | 49 (24.1) | 53 (26.6) | 58 (29.4) |
| Don’t know | 17 (8.5) | 14 (6.9) | 12 (6.1) | 14 (7.1) |
| Not declared | 4 (1.9) | 5 (2.5) | 5 (2.5) | 1 (0.5) |
| Housing, *n* (%) |  |  |  |  |
| Private | 141 (70.2) | 157 (77.3) | 144 (72.4) | 144 (73.1) |
| Public | 54 (26.9) | 40 (19.7) | 48 (24.1) | 48 (24.4) |
| Other | 2 (1.0) | 1 (0.5) | 2 (1.0) | 4 (2.0) |
| Not declared | 4 (1.9) | 5 (2.5) | 5 (2.5) | 1 (0.5) |

**Accelerometer Profiles and Baseline Measures of Outcome Variables by Intervention Groups**

|  | Control (*n*=201) | Fitbit Only (*n*=203) | Charity (*n*=199) | Cash (*n*=197) | p value^a^ |
| --- | --- | --- | --- | --- | --- |
| Valid participants^b^, *n* (%) |  |  |  |  |  |
| Baseline | 186 (92.5%) | 197 (97.0%) | 193 (97.0%) | 197 (100.0%) | <.001 |
| M6 (6 months) | 177 (88.1%) | 175 (86.2%) | 170 (85.4%) | 184 (93.4%) | .060 |
| M12 (12 months) | 163 (81.1%) | 153 (75.4%) | 147 (73.9%) | 151 (76.7%) | .352 |
| Baseline measures, Mean (*SD*) |  |  |  |  |  |
| Daily wear time (min/day) | 865.2 (93.5) | 846.9 (72.5) | 857.1 (97.1) | 851.1 (83.4) | .190 |
| Total ST (min/day) | 617.2 (98.4) | 601.1 (80.2) | 613.0 (98.7) | 603.5 (84.5) | .254 |
| 30-min PSB (min/day) | 233.8 (101.8) | 220.6 (94.3) | 222.3 (98.5) | 212.5 (85.5) | .182 |
| Daily step counts (steps/day) | 8031.9 (2501.3) | 8035.0 (2359.6) | 7776.9 (2256.0) | 8298.2 (2499.7) | .202 |

*Note*. ST = sedentary time. PSB = prolonged sedentary bouts.

^a^ *p* values were obtained using a chi-square test of independence for categorical variables and general linear models for continuous variables. ^b^ valid participant was defined as having 4 or more valid days (10 or more wear hours) including one weekend day.
